# Supplementary material for: Comparative Transcriptomic Analysis of the Pituitary Gland between Cattle Breeds Differing in Growth: Yunling Cattle and Leiqiong Cattle
Source: Animals (Basel). 2020 Jul 25;10(8):1271. doi: 10.3390/ani10081271 (PMC7460210; doi:10.3390/ani10081271)
Supplement: Supplementary file 1 [file animals-10-01271-s001.zip › supplementary files/Table S1 Specific primers used for qRT-PCR.docx]

**Supplementary Table 1.** Specific primers used for qRT-PCR.

| Genes | Primers (5′–3′) | Product length | Accession number^1^ |
| --- | --- | --- | --- |
| GHRHR | F: ATCATCAAAGGACCCATC | 76bp | XM_010804289 |
|  | R: TCAGCAGGATACGGATAA |  |  |
| JAG1 | F: AGACACTGAAGCAGAACA | 83bp | NM_001191178 |
|  | R: GCCGAAGCCATAGTAATAG |  |  |
| FAM92B | F: GCTAAAGGACATTCAGAAGA | 91bp | NM_001077134 |
|  | R: GAAGGCACTGGAATACAC |  |  |
| SRMS | F: TGCTCTACGAGGTCTTCAC | 80bp | NM_001192544 |
|  | R: CCGTGCGATCTGCTGTAG |  |  |
| OPRK1 | F: CTTCATCTGTTGGCATCT | 105bp | NM_001046480 |
|  | R: AGGAGTAGTCGTCATCTG |  |  |
| NELL2 | F: CAAGTGCTGTAAGGAATG | 110bp | NM_001102084 |
|  | R: ACTCATAGAGAACACATACT |  |  |
| FNIP2 | F: AAGAAGATTGCCATAAGC | 114bp | XM_015475420 |
|  | R: CCTGTTCATGTGAGATTC |  |  |
| SYTL2 | F: AACGACTTCTGGTTCCTT | 104bp | NM_001102278 |
|  | R: TGTGTTCATTGATGGTGTG |  |  |
| CXCL14 | F: GAGGAGAAGATGGTTATC | 110bp | NM_001034410 |
|  | R: TTGTACCATTTGATGAAC |  |  |
| HTR3E | F: AATTCATGGATGTGGATAAG | 119bp | XM_005201627 |
|  | R: GTAGAAGATGTTCAGGTTAC |  |  |
| GAPDH | F: GCATCGTGGAGGGACTTATGA | 67bp | NM_001034034.2 |
|  | R: GGGCCATCCACAGTCTTCTG |  |  |
| RPL4 | F: AGGAGGCTGTTCTGCTTCTG | 185bp | NM_001014894.1 |
|  | R: TCCAGGGATGTTTCTGAAGG |  |  |
| RPL11 | F: CCTCTTCCTTCCGGCTGTC | 279bp | NM_001206932 |
|  | R: TCCTGGTCTGTTCCTTCACA |  |  |

^1^From the database of the National Center for Biotechnology Information
